# Real world data from a multi-centre study on the effects of cilostazol on pain symptoms and walking distance in patients with peripheral arterial disease

**DOI:** 10.1186/s13104-022-06264-0

**Published:** 2022-12-20

**Authors:** Niki Katsiki, Nikolaos Tentolouris, Georgios Marakomichelakis, Dimitrios Richter, Athanasios Giannoukas, Panagiota Koufaki, Nikolaos Papanas, CI-WIN Investigators, Ilias Alexopoulos, Filippos Anastasiadis, Evangelos Andreadis, Georgios Andreopoulos, Dimitris Apostolidis, Ioannis Christodoulou, Ioannis Christopoulos, Eleftherios Dalaberis, Hermioni Daliani, Giorgos Dimitriou, Iordanis Dimitsikoglou, Nikolaos Dimoulis, Dimitrios Doulgerakis, Ioannis Douloumpakas, Efrosyni Drakopoulou, Theodoros Felekis, Theodoros Feloukas, Sofia Florou, Dimitrios Fragakis, Theodoros Fregidis, Panagiotis Gakis, Christos Galanakis, Antonios Giakoumis, Nikolaos Giannes, Alexandros Galapis, Ioannis Gouveris, Theodoros Groutsis, Vasileios Grapsas, Panagiotis Grigoropoulos, Triantafyllos Iordanidis, Kyriakos Kazakos, Maria Kazantzi, Haralabos Kapernopoulos, Konstantinos Kapetanios, Eleni Karagianni, Fotis Karakostas, Dimitris Karapiperis, Antonios Karotsis, Abraam Karsanidis, Fotios Kasfikis, Spyridon Kourouklis, Tsampikos Kourtis, Paraskevi Kourtidou, Stylianos Koutsias, Stavros Kotsogiannis, Ioannis Lampousakis, Panagiotis Latsios, Irine Liouri, Panagiotis Makrygiannis, Welcome Matsouki, Konstantina Bakalakou, Pavlos Bakiris, Maria Balogianni, Apostolos Benis, Vasiliki Beri, Antonios Beroukas, Konstantinos Moysidis, Efstathios Nanos, Achilleas Nikolaou, Marina Dubovina, Nikolaos Oikonomidis, Emmanouil Pangalos, Georgios Panagoulias, Savvas Papadopoulos, Georgios Paraskevas, Michael Peroulis, Vasilis Petoumenos, Spyridon Petrogiannis, Georgios Pournaras, Helena Pourou, Markos Prionidis, Antonios Rigas, Spyridon Rigas, Dimitrios Rigopoulos, Dimitrios Sakellariou, Periklis Sarafianos, Konstantinos Svolis, Maria Seferou, Vasiliki Sklirou, Georgios Spais, Christos Stathopoulos, Victoria Stavridou, Ioannis Stavrou, Ioannis Sfiniadakis, Vagia Tatola, Isaak Topalidis, Aikaterini Trikkalinou, Dimitrios Tsagarakis, Panagiotis Tsarouchas, Georgios Valkaniotis, Maria Varella, Stavroula Vasilakakou, Maria Vlachopoulou, Antonios Vouzas, Ioannis Zafeiriou, Ioannis Zafeiris, Ourania Zacharopoulou, Dimosthenis Zinelis

**Affiliations:** 1 Department of Nutritional Sciences and Dietetics, International Hellenic University, Thessaloniki, Greece; 2 School of Medicine, European University Cyprus, Nicosia, Cyprus; 3 First Department of Propaedeutic and Internal Medicine, Medical School, National and Kapodistrian University of Athens, Athens, Greece; 4 Fourth Department of Internal Medicine and Angiology Unit, Evangelismos General Hospital, Athens, Greece; 5 Cardiac Department, Euroclinic Hospital, Athens, Greece; 6 Department of Vascular Surgery, University Hospital of Larissa, Faculty of Medicine, School of Health Sciences, University of Thessaly, Larissa, Greece; 7 WINMEDICA Hellas, Athens, Greece; 8 Diabetic Foot Clinic, Diabetes Centre, Second Department of Internal Medicine, University Hospital of Alexandroupolis, Democritus University of Thrace, Alexandroupolis, Greece

**Keywords:** Cilostazol, Peripheral arterial disease, Intermittent claudication, Pain-free walking distance

## Abstract

**Objective:**

to assess the effects of cilostazol on pain-free walking distance in PAD patients with IC at 3 and 6 months in a real world, prospective, observational study. We included 1015 PAD patients presenting with IC (71.3% men, 93.5% white, mean age 69.2 ± 8.7 years). Patients were followed up for 6 months by their physicians.

**Results:**

Cilostazol significantly increased pain-free walking distance by a median of 285 and 387 m at 3 and 6 months, respectively (p < 0.01 for all comparisons). This effect was significant for patients 50–74 years (but not for those aged ≥ 75 years) and independent of smoking status, changes in physical activity, comorbidities and concomitant medication for PAD (i.e., acetylsalicylic acid and clopidogrel). Furthermore, significant reductions were observed in systolic (from 139 ± 16 to 133 ± 14 mmHg; p < 0.001) and diastolic blood pressure (from 84 ± 9 mmHg to 80 ± 10 mmHg; p < 0.001). Smoking cessation and increased physical activity were reported by the majority of participants. In conclusion, cilostazol was shown to safely decrease pain symptoms and improve pain-free walking in PAD patients with IC in a real world setting. Benefits also occurred in terms of BP and lifestyle changes.

## Introduction

Peripheral artery disease (PAD) is linked with increased cardiovascular (CV) risk, limb morbidity and all-cause death [1]. Age, smoking and type 2 diabetes mellitus (T2DM) are strong predictors of PAD development [2–5]. In all PAD patients, best medical therapy (BMT) should be implemented, including best pharmacological therapy (antihypertensive, lipid-lowering and antithrombotic drugs), as well as smoking cessation, healthy diet, weight loss and regular physical exercise [6]. Unfortunately, PAD frequently remains undiagnosed, and thus untreated, due to the absence of typical clinical symptoms (i.e., intermittent claudication, IC), as well as the lack of disease awareness for both patients and physicians [5]. In the presence of IC, the risk of CV and limb morbidity, as well as all-cause mortality, are further increased [5]. Hence, PAD treatment should be immediately initiated, targeting at controlling CV risk factors and improving IC.

Cilostazol is a unique antiplatelet drug that selectively targets phosphodiesterase III (PDE-III), and thus, apart from inhibiting platelet aggregation (induced by epinephrine, collagen, arachidonic acid and 5′-adenosine diphosphate), it can also improve endothelial cell function [6]. Cilostazol-induced PDE-III inhibition primarily increases cAMP levels, subsequently leading to upregulation of protein kinase A (PKA) activity, which phosphorylates key molecules in the process of platelet aggregation [7]. Cilostazol-mediated increase in cAMP levels also involves the inhibition of adenosine re-uptake, resulting in raised circulating and interstitial adenosine levels, which, in turn, bind to adenosine receptors [8]. Thus, adenylate cyclase activity is upregulated via Gs proteins [8]. In addition to PDE-III, cilostazol inhibits the activity of the multidrug resistance protein 4 (MRP4), which is implicated in platelet aggregation [9] and residual platelet reactivity following aspirin therapy [10].

Potential mechanisms for the vasodilatory effect of cilostazol include PKA phosphorylation of myosin light chain kinase, transient receptor potassium canonical channels, endothelial nitric oxide synthase (eNOS) and G protein coupled receptor kinase 2, as well as hyperpolarisation of smooth muscle cell membranes and inactivation of Galpha-q-mediated signalling [8]. In relation to its antiproliferative actions, cilostazol downregulates (via cAMP elevation and PKA activation) several endothelial adhesion molecules, such as the vascular cell adhesion molecule (VCAM), the intercellular adhesion molecule (ICAM) and E-selectin, as well as modulates growth factors [e.g. platelet-derived growth factor (PDGF), vascular endothelial growth factor (VEGF) and nitric oxide] [8] Of note, cilostazol upregulates eNOS activity (thus, improving endothelial dysfunction) through multiple pathways, including activation of PKA and kinase Akt [8].

Cilostazol has been reported to increase pain-free walking distance in PAD patients with IC, as supported by previous Cochrane reviews, dated in 2008 and 2014 [8, 11–13] and an updated one, published in 2021 [14]. Indeed, cilostazol is mainly indicated for improving IC in PAD patients, although it may also exert other “pleiotropic” CV effects (e.g. antithrombotic, vasodilation, inhibition of vascular smooth muscle cell proliferation, protection from restenosis) [15]. Such cilostazol-induced CV benefits may lead to better limb and CV outcomes, but strong evidence is lacking in this field [14, 16–19]. Real-world data are important in addressing major public health problems, such as PAD [20]. Furthermore, such data provides a “realistic” view of how (and if) a disease is managed in daily practice, as well as the level of clinical guidelines implementation. Therefore, we aimed to examine the effects of cilostazol on pain-free walking distance in PAD patients with IC, as well as to record how PAD is treated in primary care in a real world, prospective, observational study.

## Main text

### Patients and methods

This is a non-interventional, observational, phase 4, trial in PAD patients presenting with IC. The study aimed to assess the safety and efficacy of cilostazol in improving pain symptoms and walking distance at 3 and 6 months from cilostazol therapy initiation. Lifestyle measures and drug treatment were also recorded during the follow-up.

Inclusion criteria were: age ≥ 50 years, Fontaine Stage II PAD (IC), current cilostazol treatment and ankle-brachial index (ABI) 0.41–0.9 (if available). Exclusion criteria were: known hypersensitivity to cilostazol; creatinine clearance ≤ 25 ml/min; moderate or severe hepatic dysfunction; pregnancy; participation in another clinical trial; known predisposition to bleeding (e.g. recent [within 6 months] haemorrhagic stroke, active peptic ulcer, poorly controlled hypertension, hyperplastic diabetic retinopathy); congestive heart failure; history of ventricular fibrillation or ventricular tachycardia; multifocal abdominal dislocations; QTc prolongation; history of severe tachyarrhythmia; concomitant treatment with ≥ 2 additional antiplatelet or anticoagulant agents (e.g. acetylsalicylic acid, clopidogrel, acenocoumarol, warfarin, heparin, dabigatran, apixaban or rivaroxaban); unstable angina pectoris, myocardial infarction or coronary intervention in the last 6 months.

The study involved 101 sites (hospital and private practice) in Greece, under the co-ordination of 5 hospital centres, between May 2016 and November 2017. Patient care was provided as per usual practice by the attending physicians and all patients were followed during 3 visits: first, second and third visits performed at baseline, 3 and 6 months, respectively. In each visit, medical history, blood pressure, current drug treatment, smoking status, physical activity, symptoms of IC and pain-free walking distance were recorded. The latter was mostly self-reported (i.e., without treadmill use).

The study was performed in accordance with the current version of the EU Regulations (Clinical Trial Regulation EU No. 536/2014) and it was conducted in agreement with the International Conference on Harmonisation (ICH) guidelines for Good Clinical Practise (GCP), while following the guidelines of the Greek National Organisation for Medicines (EOF). The study was approved by the Institutional Review Boards (Hospital Ethics Committees) of all co-ordinating Greek Hospitals (University Hospital of Alexandroupolis; Papageorgiou General Hospital of Thessaloniki; Evangelismos General Hospital, Athens; Euroclinic Hospital, Athens; University Hospital of Larissa; General Hospital ‘‘Laiko’’, Athens). All patients had signed written informed consents before study enrollment.

### Statistical analysis

Qualitative data was presented as percentages, whereas quantitative data was presented as mean ± standard deviation (SD) or median (range) for non-parametric variables. Distribution of qualitative variables was compared using the χ^2^ test, whereas, the one-way ANOVA test (or the non-parametric Kruskal Wallis test) was used to compare variances of quantitative variables. Statistical analyses were performed using STATISTICA version 13.0 PL (Dell Inc, Texas, USA). A 2-sided p < 0.05 was considered significant.

According to sample size calculation, to achieve a confidence level of 99.9% and a statistical error threshold of p < 0.05 in the study’s results, a sample size of at least 1000 PAD patients was required.

## Results

The study included 1015 patients (71.3% men, 93.5% white) with a mean age of 69.2 ± 8.7 years. Of these, 28% of patients were < 65 years, 41.4% were between 65 and 74 years and 30.6% were ≥ 75 years of age. In relation to smoking status, 40.2% were current, 32.3% former and 27.5% non-smokers. Furthermore, 53.7% had T2DM.

During cilostazol therapy, significant reductions were observed in both systolic (from 139 ± 16 mmHg at baseline to 133 ± 14 mmHg at 6 months; p < 0.001) and diastolic BP (from 84 ± 9 mmHg at baseline to 80 ± 10 mmHg at 6 months; p < 0.001). Of note, these reductions occurred without any modifications in the antihypertensive drug treatment of the patients (Table 1). With regard to PAD drug treatment, the percentage of patients on 100 mg cilostazol significantly increased (from 53.8% at baseline to 67.5% at 6 months; p < 0.01), whereas those on 50 mg cilostazol significantly decreased (from 45.9% at baseline to 32.5% at 6 months; p < 0.01) (Table 2). Furthermore, both acetylsalicylic acid and clopidogrel use significantly increased during follow up (acetylsalicylic acid: from 20.9% at baseline to 32.8% at 6 months; p < 0.01-clopidogrel: from 14.8% at baseline to 22.7% at 6 months; p < 0.01) (Table 2).

**Table 1:** Changes in blood pressure, low-density lipoprotein cholesterol, antihypertensive and hypolipidemic treatment during study follow-up

	Visit I (baseline) (n = 1015)	Visit II (3 months) (n = 1015)	Visit III (6 months) (n = 1015)	P value
SBP [mmHg]	139 ± 16	136 ± 12	133 ± 14	< 0.001
DBP [mmHg]	84 ± 9	82 ± 8	80 ± 10	< 0.001
Hypolipidemic drugs [n (%)]				
Statins	605 (59.6%)	609 (61.8%)	596 (60.7%)	NS
Fenofibrate	53 (5.2%)	59 (6.0%)	58 (5.9%)	NS
Ezetimibe	89 (8.8%)	96 (9.7%)	96 (9.8%)	NS
Omega-3 lipid mediators	64 (6.3%)	62 (6.3%)	60 (6.1%)	NS
Other	3 (0.3%)	3 (0.3%)	3 (0.3%)	NS
Antihypertensive drugs [n (%)]				
Calcium channel blockers	399 (39.3%)	392 (39.8%)	386 (39.3%)	NS
Diuretic	372 (36.7%)	371 (37.7%)	372 (37.9%)	NS
Angiotensin receptor blockers	245 (24.1%)	239 (24.3%)	235 (23.9%)	NS
Angiotensin II receptor blockers	381 (37.5%)	379 (38.5%)	371 (37.8%)	NS
Beta blockers	225 (22.2%)	221 (22.4%)	218 (22.2%)	NS

*SBP* systolic blood pressure, *DBP*: diastolic blood pressure, *LDL-C* low-density lipoprotein cholesterol, *NS* non-significant

**Table 2:** Drug therapy for peripheral artery disease and lifestyle changes

	Visit I (baseline) (n = 1015) %	Visit II (3 months) (n = 1015) %	Visit III (6 months) (n = 1015) %	P value
Treatment for intermittent claudication [n (%)]				
50 mg cilostazol	466 (45.9)	337 (34.2)	319 (32.5)	< 0.01
100 mg cilostazol	546 (53.8)	648 (65.8)	663 (67.5)	< 0.01
Pentoxifylline	14 (1.4)	0 (0)	0 (0)	–
Acetylsalicylic acid	212 (20.9)	318 (32.3)	322 (32.8)	< 0.01
Clopidogrel	150 (14.8)	224 (22.7)	223 (22.7)	< 0.01
Lifestyle change [n (%)]				
Smoking cessation	0 (0.0)	768 (78.0)	809 (82.4)	< 0.01
Exercise increase	0 (0.0)	742 (75.3)	769 (78.3)	< 0.01

At 6 months, 82.4% of smokers reported smoking cessation. Furthermore, 78.3% of the overall patient population reported increased physical activity (Table 2).

Overall, cilostazol therapy resulted in an increase in pain-free walking distance by a median of 285 m at 3 months and 387 m at 6 months (p < 0.01 for all comparisons). This effect was significant for patients 50–74 years (but not for those aged ≥ 75 years) and independent of smoking status, changes in physical activity, comorbidities and PAD drug co-treatment (i.e. acetylsalicylic acid and clopidogrel) (Table 3).

**Table 3:** Changes in pain free walking distance during follow up, according to age, physical activity, comorbidities and drug co-treatment

	n	Visit I (baseline)	Visit II (3 months)	Visit III (6 months)	p-value
According to age					
50–64 years	285	346 (312–380)	640 (594–687)	819 (759–878)	0.047
65–74 years	487	275 (253–296)	569 (538–600)	761 (720–802)	< 0.02
≥ 75 years	243	239 (208–269)	510 (472–548)	671 (622–720)	NS
According to smoking status					
Former smokers	542	300 (244–363)	514 (456–599)	608 (578–692)	< 0.03
Current smokers (at 6 months)	143	259 (160–340)	420 (448–579)	581 (543–673)	< 0.03
Non-smokers	271	265 (233–296)	543 (508–577)	728 (678–779)	< 0.04
According to changes in physical activity					
In patients with no increase in physical activity	212	262 (242–282)	499 (453–544)	703 (645–761)	< 0.05
In patients who have increased their physical activity during follow up	542	274 (252–295)	555 (525–585)	750 (711–789)	< 0.02
According to comorbidities					
In the presence of hypertension	873	288 (270–306)	563 (540–586)	740 (710–769)	< 0.02
In the presence of type 2 diabetes	545	266 (247–284)	542 (512–571)	712 (672–751)	< 0.03
In the presence of dyslipidaemia	108	331 (269–393)	563 (509–617)	800 (706–893)	< 0.03
According to PAD drug co-treatment					
Cilostazol 100 mg monotherapy	328	345 (311–379)	650 (603–698)	832 (782–883)	< 0.01
Cilostazol 50 mg monotherapy	249	298 (264–331)	646 (600–693)	791 (738- 843)	< 0.03
Cilostazol 50 mg and acetylsalicylic acid	126	358 (301–415)	599 (534–664)	742 (652–831)	< 0.04
Cilostazol 100 mg and acetylsalicylic acid	97	233 (188–279)	569 (488–650)	836 (709–964)	< 0.05
Cilostazol 50 mg and clopidogrel	80	255 (221–290)	513 (442–583)	742 (640–845)	< 0.04
Cilostazol 100 mg and clopidogrel	135	279 (235–323)	539 (468–609)	711 (626–796)	< 0.03

*NS* non-significant, *PAD* peripheral artery disease

Cilostazol use was well-tolerated. Adverse events (AEs) included headache, diarrhoea and nausea. These events were generally of mild to moderate severity, transient or resolved after symptomatic treatment, and they did not require treatment discontinuation. Cilostazol did not increase the risk of bleeding, even in patients taking background antiplatelet or anticoagulant therapy.

## Discussion

In the present study among 1015 PAD patients with IC, cilostazol significantly increased pain-free walking distance by 285 m at 3 months and 387 m at 6 months, irrespective of lifestyle changes and comorbidities. This finding is in agreement with previous evidence from Cochrane reviews [12–14] and guidelines [10]. The benefit was also observed, but lost its significance, in the older PAD patients (i.e., aged ≥ 75 years). However, it should be noted that this age group was smaller (n = 243) than those aged 50–64 (n = 285) and 65–74 years (n = 487), thus possibly affecting the results.

Interestingly, at the end of the study, 82.4% of smokers reported quitting smoking and 78.3% of all PAD patients reported increased physical activity. Smoking cessation and exercise are strongly recommended for PAD treatment by both the American Heart Association (AHA) [20] and the European Society of Cardiology (ESC) [11]. Therefore, these findings potentially highlight the importance of “real-world” recordings in improving disease awareness (for both physicians and patients), as well as implementation of guidelines (for physicians) and adherence to treatment (for patients).

In the present study, BP was significantly decreased following cilostazol therapy. It should be noted that antihypertensive drug therapy remained unchanged during follow-up. Cilostazol can reduce BP (due to its vasodilating properties [10]), as also reported in its summary of product characteristics [21].

The effects of cilostazol on LDL-C have not been well studied yet. In a previous study, no changes in LDL-C levels were observed among 189 PAD patients with IC treated with cilostazol for 12 weeks [22]. Similar results were reported in 17 PAD patients with T2DM [23]. Regrettably, no lipid values were available in this study.

Impressively, smoking cessation and increased physical activity were observed in the present study. These novel findings merit replication and further study. If confirmed, they may be taken to increase the benefits of cilostazol in clinical reality.

Cilostazol was safe and well tolerated. No treatment discontinuation was reported. This agrees with its overall safety profile [12–14] and guidelines [11].

## Conclusions

Cilostazol was shown to safely decrease pain symptoms and improve pain-free walking in PAD patients with IC in a real world setting. Benefits also occurred in terms of BP and lifestyle changes. Further research is needed to establish the impact of cilostazol therapy on other cardiometabolic parameters. Overall, public (and physician) awareness on PAD should be increased to ensure early detection and adequate treatment (both lifestyle modifications and drug therapy).

## Strengths and limitations

The strengths of this study include its relatively large number of participants and its real-world setting. There are certain limitations, as well. First, pain-free walking distance was mostly self-reported, since treadmill was not available in the majority of the centres. Secondly, several cardiometabolic laboratory parameters were not recorded and thus were unavailable for further analyses. However, the present study was large enough to reach safe conclusions in relation to its main objective, i.e., the efficacy of cilostazol in improving IC and increasing pain-free walking in PAD patients.

## Data Availability

Data is available upon reasonable request.

## Abbreviations

ABI: Ankle-brachial index
AHA: American Heart Association
CV: Cardiovascular
ESC: European Society of Cardiology
IC: Intermittent claudication
PAD: Peripheral arterial disease
T2DM: Type 2 diabetes mellitus
